# Pharmacokinetic studies in children: recommendations for practice and research

**DOI:** 10.1136/archdischild-2017-314506

**Published:** 2018-04-19

**Authors:** Charlotte I S Barker, Joseph F Standing, Lauren E Kelly, Lauren Hanly Faught, Allison C Needham, Michael J Rieder, Saskia N de Wildt, Martin Offringa

**Affiliations:** 1 Infection, Inflammation and Rheumatology Section, UCL Great Ormond Street Institute of Child Health, University College London, London, UK; 2 Paediatric Infectious Diseases Research Group, Institute for Infection and Immunity, St George’s University of London, London, UK; 3 Paediatric Infectious Diseases Unit, St George’s University Hospitals NHS Foundation Trust, London, UK; 4 Department of Pediatrics and Child Health, University of Manitoba, Winnipeg, Manitoba, Canada; 5 Clinical Trials Platform, George and Fay Yee Centre for Healthcare Innovation, Winnipeg, Manitoba, Canada; 6 Departments of Paediatrics, Physiology and Pharmacology and Medicine, Western University, London, Ontario, Canada; 7 Molecular Medicine Group, Robarts Research Institute, London, Ontario, Canada; 8 Child Health Evaluative Sciences, The Hospital for Sick Children, Toronto, Ontario, Canada; 9 Department of Pharmacology and Toxicology, Radboud University, Nijmegen, The Netherlands; 10 Intensive Care and Department of Pediatric Surgery, Erasmus MC Sophia, Rotterdam, The Netherlands; 11 Division of Neonatology, Department of Paediatrics, University of Toronto, Toronto, Ontario, Canada

**Keywords:** pharmacology, dosing, pharmacometrics, paediatrics, neonatology

## Abstract

Optimising the dosing of medicines for neonates and children remains a challenge. The importance of pharmacokinetic (PK) and pharmacodynamic (PD) research is recognised both in medicines regulation and paediatric clinical pharmacology, yet there remain barriers to undertaking high-quality PK and PD studies. While these studies are essential in understanding the dose–concentration–effect relationship and should underpin dosing recommendations, this review examines how challenges affecting the design and conduct of paediatric pharmacological studies can be overcome using targeted pharmacometric strategies. Model-based approaches confer benefits at all stages of the drug life-cycle, from identifying the first dose to be used in children, to clinical trial design, and optimising the dosing regimens of older, off-patent medications. To benefit patients, strategies to ensure that new PK, PD and trial data are incorporated into evidence-based dosing recommendations are needed. This review summarises practical strategies to address current challenges, particularly the use of model-based (pharmacometric) approaches in study design and analysis. Recommendations for practice and directions for future paediatric pharmacological research are given, based on current literature and our joint international experience. Success of PK research in children requires a robust infrastructure, with sustainable funding mechanisms at its core, supported by political and regulatory initiatives, and international collaborations. There is a unique opportunity to advance paediatric medicines research at an unprecedented pace, bringing the age of evidence-based paediatric pharmacotherapy into sight.

## Introduction

The importance of improving pharmacotherapy for children is widely recognised. Until recently, only 30% of medications used in paediatrics in the USA were actually studied in children.^1^ The lack of appropriate drug labelling for children is a long-standing problem,^2^ which also has implications for drug safety, particularly in neonates.^3^ High-quality pharmacokinetic (PK) and pharmacodynamic (PD) studies are an essential component of understanding the dose–concentration–effect relationship and should underpin dosing recommendations in children. Without PK data from appropriate populations to support marketing authorisations, clinicians have little choice but to use medications off-label, which can lead to increased risk of toxicity or subtherapeutic dosing. During drug development, model-based (pharmacometric) approaches can be used in several ways, including identifying the dose regimen for different age groups in order to inform clinical trial design. PK/PD models can inform the expected effect size during sample size calculations, reducing the burden of unnecessary research, and also help refine dosing strategies relevant to routine clinical practice.

Despite detailed guidance on adult PK/PD research, specific challenges affect the design and conduct of paediatric pharmacological studies. This review defines the key challenges encountered, exploring solutions and providing recommendations for practice and future research.

### Paediatric PK studies: what are they?

PK studies absorption, distribution, metabolism and excretion of drugs. PK studies guide the determination of a drug’s optimal route of administration and formulation, and appropriate dose and dose schedule(s). PK studies include (1) early-phase studies including PK components, comprising (a) first-dose-in-child studies and (b) phase II and III studies; and (2) phase IV postmarketing PK studies (including, eg, dose optimisation or therapeutic drug monitoring (TDM) studies).

Paediatric research is challenging because children are considered vulnerable and sample sizes are small. Nevertheless, accurate dosing is paramount to safe and effective treatment, which makes paediatric PK studies essential. The USA and European Union (EU) have adopted paediatric clinical trial regulations to address historic deficiencies in this research.^4^ These reflect the recent paradigm shift in attitudes recognising that ‘the time has come to protect children and young people *through* research not *from* research’.^5^

### Knowledge gaps in developmental physiology and pharmacology

Physiological development, including physical growth and the maturation of organs, transporters and enzymes, creates size and age-dependent variability in PK parameters. PK measures should therefore be related to measures of growth, including age, weight or body surface area (BSA).^6^ The heterogeneity in paediatric patient populations contributes to large PK variability: neonates, for example, can have a tenfold weight range (eg, 400 g–4 kg) in addition to differing gestational ages and postmenstrual ages. Critical illness, renal replacement therapy and cardiopulmonary bypass can also influence PK, as well as drug–drug interactions.^7^ Furthermore, genetic differences (polymorphisms) contribute to variability in drug disposition and response. A deeper understanding of the interplay between genetic polymorphisms, growth and development is urgently needed to be able to provide personalised dosing to children of different ages, underlying disease states and treatments.^8^

### Challenges in paediatric PK

Traditional PK study designs involve a single dose with multiple blood samples at fixed intervals, covering a time period of up to approximately 10 half-lives. Multiple samples per patient, with varying routes of administration, enable calculation of individual drug exposure (area under the plasma drug concentration–time curve: AUC), and the rate and extent of absorption. Such studies generally require the same number of samples from all subjects taken at the same time in the dosing interval. These designs present ethical and practical barriers for children. Similar challenges arise later in the drug life-cycle when needing to undertake verification of bioequivalence or cross-over relative bioavailability studies in children in order to exclude age-by-formulation interactions.

First, the number of samples and the blood volume per sample that can safely be taken in children—especially neonates—are limited.^9^ Second, it is impractical to collect many samples over a long period, without interfering with patient care and length of stay. Phlebotomy-related barriers partly concern the acceptability of additional sampling in children, particularly when venepuncture is not needed for clinical care. Similar challenges arise later in the drug life-cycle when needing to undertake verification of bioequivalence or cross-over relative bioavailability studies in children in order to exclude age-by-formulation interactions.

In neonatal studies, differences in postmenstrual age, body composition and end-organ perfusion create extensive PK variability, reflecting rapid phases of growth and maturation. Usually, prior to neonatal PK studies, evidence should be established in less vulnerable populations.^3^ However, particularly in conditions unique to newborns, neonatal PK data should be obtained a priori.^3^ Current regulations stipulate that the volume of blood samples for research should not exceed 1% at any one time (0.8 mL/kg) or 3% within 1 month, that is, 1.2 mL for a neonate weighing 500 g, which is problematic for many assays.^3 10^

### Sampling techniques for blood and other biological matrices

Advances in analytical technology have dramatically reduced the blood volume required, yet novel biological sampling methods have been relatively underused. Microsampling techniques (eg, dried blood spots) are now possible with ultrasensitive analytical methods (eg, liquid-chromatography with tandem mass-spectrometry), potentially reducing sample volumes to 5–10 μL, sometimes also reducing invasiveness and improving analyte stability.^10^

A thorough understanding of different paediatric blood sampling techniques, summarised in table 1, is paramount for PK study design. Research methods which reduce the burden of blood sampling, including so-called ‘scavenged’ sampling^11^ (using left-over blood from clinical samples) or opportunistic sampling (sampling at the same time as clinical blood tests), can be used but also require careful validation, to ensure they provide equivalent estimation of PK parameters when compared to preplanned sampling time points.^12^

**Table 1:** Paediatric blood sampling procedures that may be employed in pharmacokinetic studies or clinical trials

Method of blood sampling	Advantages	Disadvantages	Preferred age group(s)	Comments
Sampling from an indwelling arterial catheter or central venous line	No need for separate invasive procedures (that would require additional needles). If such a line is already in place for clinical purposes, the risks associated with blood sampling are limited.	Potential infection risk from additional accessing of the line; blood loss due to inappropriate line handling; premature loss of the line. Sometimes additional blood volume (dead-space) needed to clear the line of other infusion fluids.	Method feasible in all age groups.	Some centres return this dead-space volume directly after sampling, while others consider it unhygienic, dependent on the structure of the specific line system used and local practice. Method commonly used in intensive care settings.
Cannulation-based venepuncture	Different options are possible, either multiple or single use of an intravenous cannula. With multiple use of a single intravenous cannula, the burden of the insertion is reduced to only once.	Often multiple attempts are needed before successful peripheral cannulation. The blood flow may be too slow and blood may clot in the cannula system, even when intermittent or continuous saline flushes are instilled in the cannula.	For smaller children, open collection systems are more appropriate.	In very small children, repeated sampling from one cannula may also be difficult.
Venepuncture (without cannulation)—(1) vacuum systems	Several methods can be used: in older children, simple vacuum systems in large veins are most frequently employed. Discomfort can be reduced by appropriate use of local anaesthetic cream.	Usually involves study-specific invasive procedure. Less suitable for younger children. Not suitable for some children who have experienced very high numbers of routine blood tests (eg, in oncology).	Preferred in older children. In younger infants and neonates, these methods are less practical or even unfeasible, as the size of the vein means the vacuum will collapse the vessel so no blood can be taken.	Culturally specific factors can be important: parents in some countries are happy for their children to have extra blood tests at any age, whereas other cultures can be very against the invasiveness of the method.
Venepuncture—(2) non-vacuum methods: for example, the use of syringe needles or the needle from a Vacutainer system	These needles are easier to insert and manipulate in small veins than intravenous cannulae and have less problems with blood clotting, due to the large bore size (syringe needles) and heparin coating (Vacutainer needles).	This method needs practice by specifically trained personnel.	Preferable for younger children (in whom vacuum-based systems and/or cannulation may be difficult).	In all these non-vacuum methods, blood needs to drop in opened tubes. In general blood samples up to 5 mL per occasion can be taken, before the blood starts to clot, but it is widely variable per patient and becomes less with decreasing age.
Capillary sampling: heel prick or finger prick	The advantage of finger/heel pricks is that they can be easily taught to parents and children. This method can be less invasive and painful than venepuncture or intravenous cannulation (although this is debatable).	Capillary sampling is not always comfortable: studies have shown that venepuncture is preferred over finger pricks in older children. Also, to obtain adequate blood volumes, repeated punctures may be needed, and also continued pressing of the foot or fingers, which is uncomfortable.	The heel prick method is often preferred in neonates when normal venepuncture and cannulation are not required for clinical reasons.	Since this can be taught to children or their carers, they may be able to collect blood samples at home, in connection with dried blood spot analysis.

The collection of alternative matrices to blood requires special consideration in children. Whenever possible, PK/PD information should be obtained via less invasive matrices such as urine and saliva. If an indwelling bladder catheter is present and a reliable quantitative assay available, urine samples can also be obtained to minimise necessary blood sampling.^3^ Limitations of these approaches include analytical complexity, potential impact of collection methods and environmental factors (eg, temperature and pH) on analyte recovery, and practical challenges. Urinary sampling methods are summarised in table 2. The urine output must be collected over a prolonged (prespecified) period to measure cumulative drug (or metabolite) excretion per unit time, but collection periods are often logistically challenging in practice, deviating from clinical routines.

**Table 2:** Urinary sampling methods in pharmacokinetic (PK) studies

Methods	Pros and cons
Midstream urine sample	Feasible for older children who can follow instructions. Not suitable for young children.
Urinary catheter sampling	This method is generally limited to children with indwelling urinary catheters (IDC) for clinical care, since IDC insertion purely for research is unacceptable in most jurisdictions.
Urinary adhesive bags	These bags may seem more practical (than awkward time collection periods), but this method is notoriously unreliable. The adhesive often comes loose and urine leaks in the diaper. Repeated urine bag adhesion may damage the vulnerable skin in young infants.
Gauze-diaper methods	Urine can be collected in cotton balls or gauzes separated from the diaper interior by a plastic film. By weighing the diaper at each time interval, the total urine volume can be deduced and urine for PK analyses can be expressed from the cotton. Care must be taken to ensure that the drug is fully recovered from the urine. Also, frequent diaper changes may not be allowed in very young infants, where minimal handling and procedures are standard of care.

Saliva has shown feasibility for caffeine TDM in preterm infants^13^ but requires characterisation of the relationship between saliva and plasma concentrations. Several factors can drive variability in salivary measurements, including salivary pH and drug physicochemical properties (molecular weight, pKa). Importantly, salivary sampling in neonates can also be time-consuming, for example requiring prolonged oral cavity suctioning (up to 30 min), thereby potentially posing additional ethical concerns. Often samples from tissues of interest are more informative in understanding PK/PD, so suitable opportunities to use left-over clinical samples (eg, cerebrospinal fluid) should be taken. However, the cost of developing and validating bioanalytical assays in different sampling matrices must be incorporated into study feasibility assessments. All assays should be developed in accordance with relevant guidelines to meet regulatory requirements.^14^

### Ethical issues in paediatric PK studies

The ethical principles of paediatric research are well described elsewhere.^15^ PK research presents specific challenges surrounding the informed consent process and the risk–benefit of study participation. Potential research participants (and/or their parents) need sufficient information and time to make an informed decision. PK samples, however, may be required soon after drug administration. If this follows an emergency procedure, parents may be absent or under too much stress to consent. In these circumstances, deferred consent may be appropriate subject to ethics committee approval. For newborns, prenatal consent can be suitable.

To be ethically acceptable, participation of children should be limited to research of minimal risk and burden, or with the potential for direct benefit.^16^ Risk assessments can be difficult in paediatric studies with minimal adult safety data, for example if drug toxicity prevents healthy adult studies. Furthermore, dose-finding PK studies may not provide direct participant benefit, posing the dilemma of balancing individual research-associated burdens with intended long-term benefits to future children.

### PK modelling as a solution

To circumvent some of the challenges described above, a model-based approach to PK study design and analysis can be employed. In population PK (popPK) modelling, data from multiple subjects are analysed simultaneously, thus allowing patients to contribute data with varying sample numbers and timing. Modelling is used to estimate typical values of PK parameters for the population. Capturing information from a larger number of patients enables improved understanding of PK variability and thus improved generalisability of study results.^17^ However, PK parameters for individuals can also be estimated within the same process, making this method highly efficient. PopPK modelling using non-linear mixed effects (NLME) is now the standard method of choice. PK data (drug concentration–time profiles) are generally non-linear; hence, PK model fitting requires non-linear regression. The mixed-effects approach gives less biased and more precise estimates of variability than other methods.^18^ NLME is implemented in many general and specialist statistical tools and enables quantification of interindividual variability, intraindividual variability and residual unexplained variability. During PK model building, significant factors determining PK variability are identified (covariates, eg, age, weight, renal function). Within a mixed-effects popPK model, there are ‘fixed effects’—including the typical PK parameters, described by the PK structural model, and significant covariates—and ‘random effects’, which quantify variability at the parameter and observation levels.^17^ PK modelling confers many benefits (see box 1) and has many applications, including (1) model-based dose optimisation, (2) extrapolation for paediatric dose regimen selection, (3) clinical trial simulations, (4) model-based TDM algorithms and (5) enhancing understanding of developmental pharmacology.^17 19^

Box 1 **Key advantages of population model-based approaches to paediatric PK studies**

- Study samples do not need to be taken at exactly the same time in all patients.
- The burden of study samples can be distributed among many patients.
- Capturing data from a larger number of patients enables improved understanding of PK variability.
- Data for popPK studies can be collected in patients who are already receiving the drug of interest as part of normal clinical care.
- Optimal sampling times can be identified to improve estimation of key PK model parameters.
- Flexible study designs with sparse sampling strategies facilitate better coordination with clinical care.
- Model-based sample size calculations may incorporate prior PK data.
- Model-based analyses can evaluate additional complex factors including drug–drug interaction models, disease progression, the placebo effect and data censoring.
- Improved mechanistic understanding of drug effect (theory enrichment).
- Facilitation of extrapolation beyond observed data.
- Data synthesis: capture and integration of data from different studies.
- Hypothesis generation (during the learning phase of drug development).

PK, pharmacokinetic; popPK, population PK.

### Scaling and extrapolation

Scaling refers to normalising data either between species or between humans of different ages and sizes. PK models usually aim to estimate drug exposure (AUC), which under steady-state conditions depends only on dose and clearance (CL). In 1950, Crawford, a paediatrician, recognised CL scales approximately with BSA, and described the apparent parallel between CL scaling and that of basal metabolic rate with the so-called *allometric model*.^20^ Allometric scaling of CL a priori is now common in PK modelling and is an appropriate way to scale for size in children over 2 years of age; box 2 gives the standard allometric model.^21^

Box 2 **The standard allometric model**

$$y_{i}=a\cdot {W_{i}}^{b}$$

y_i_ is an individual’s biological parameter (eg, basal metabolic rate, drug clearance).

*a* is its value for a standard weight individual.

*W_i_* is the individual in question’s weight.

*b* is the exponent by which the parameter changes with weight.

When b is 1 there is a linear relationship between the parameter and weight, when b<1 increasing weight leads to lower proportional parameter increases, and when b>1 increasing weight leads to higher proportional parameter increases.

Delineation of the correlated effects of weight, age and organ function is challenging, generating debate regarding the appropriate value of the allometric exponent (*b)*. Knowledge from interspecies and biological process scaling, and anthropomorphic measurements (eg, organ sizes^22^), has identified 0.75 as a reasonable value for *b* when scaling CL, and 1 when scaling the volume of distribution.^21^ Weight raised to the power of 0.75 describes clearance well in children over 2 years old but may not address preceding age-related maturational effects.^23^ This can be overcome using a standard parameterisation, incorporating allometric weight scaling and a sigmoid maturation function.^6^ Germovsek *et al* 23 compared this model with distinct clearance covariate models systematically identified in the literature to fit the same data set. No published model gave a superior fit to the standard model, providing a powerful argument for researchers to adopt a standard parameterisation, facilitating comparison of studies of the same drug and model-based meta-analysis.

Renal function (as related to clearance) is a common covariate in PK studies. The Food and Drug Administration (FDA) draft guidance^24^ recommends use of the Schwartz formula in children under 12 years and the Cockroft-Gault equation in adolescents. This recommendation leads to two fundamental problems: first, the methods use demographics (eg, height, weight, age) to estimate glomerular filtration rate (GFR), which may already have entered the model (eg, via allometric scaling with a maturation function); and second, the methods have different units: mL/min/1.73 m^2^ for Schwartz and mL/min for Cockroft-Gault, respectively. One sensible solution would be to avoid GFR, and instead use serum creatinine alongside allometric scaling and a maturation function. Since serum creatinine levels change with age (high at birth, falling rapidly, then rising again), this must be scaled by the age-expected median serum creatinine; an elegant method is available.^25^ However, the contribution of muscle mass and maternal creatinine transfer in preterm neonates must be considered.^26^

*Extrapolation* is defined as ‘Extending information and conclusions available from studies in one or more subgroups of the patient population (source population) to make inferences for another subgroup of the population (target population)’.^27^ For drugs with an established linear PK profile in adults and where sufficient knowledge is available regarding disease aetiology in children, often a single-dose study generates sufficient evidence for a paediatric PK assessment.^24^ The FDA provides an algorithm (‘decision tree’) to guide appropriate extrapolation for paediatric medicines.^28^ Evidence of similar disease progression, concentration–response relationships and comparable endpoints allows the extrapolation of PK and safety studies to support paediatric dosing rationale.^28 29^ New adverse effects may subsequently be identified in children relating to developmental PD, mandating ongoing safety monitoring throughout each agent’s life-cycle.

### Dose selection for first-in-child studies: innovative methods

Often there is limited safety information to support dose selection in an initial paediatric PK study. The initial dose requires attention to (1) relative bioavailability, (2) age group and weight of study participants, (3) therapeutic index and (4) PK data from other populations and preclinical studies.^24^ Advantages of various dose-finding study designs (parallel, staggered, intrapatient dose titration) are summarised elsewhere.^29^ Current guidelines stipulate that the initial dose should be given as a fraction of the dose calculated from adult exposure based on the aforementioned factors and any additional paediatric expertise.^24^ However, the role of modelling and simulation in dose selection is increasing, to help avoid children being exposed to subtherapeutic doses using current approaches. Extrapolation can involve ‘top-down’ scaling of adult PK (described above), or ‘bottom-up’ physiology-based PK modelling, combining extensive information on the drug and the system (‘virtual child’)^30^ to estimate PK parameters. Ideally one should compare results from both approaches, with thorough investigation using sensitivity analysis to explain any differences.

To obviate potential toxicity and uncertainty surrounding first-in-child doses, some advocate microdosing (giving a very low dose), the feasibility of which has been demonstrated in infants.^31^ There are potential caveats, including limitations with assay performance and ethical concerns regarding subtherapeutic dosing. An important prerequisite is dose linearity across the range from microdose to therapeutic dose: adult studies may be required first to establish this, or a thorough theoretical evaluation. Following the first dose, Bayesian (adaptive) study designs allow dose titration to quickly reach the desired therapeutic window, within either individual or sequential patients.

### Realising an optimal design for PK studies

Optimal design refers to identification of the most informative study design, in order to optimise various aspects of the PK protocol, as summarised in box 3; results should be reviewed with consideration of practical and financial constraints.^32^

Box 3 **Features of study design that can be mathematically optimised**

- Number of PK samples required per patient.
- Timing of PK samples within the dosing interval(s).
- Number of patients within a particular age group.
- Total sample size required for robust PK parameter estimates.
- Range of covariates to be included within each cohort (eg, range of renal (dys)function).

PK, pharmacokinetic.

Many available software tools can help identify optimal study design.^33^ For paediatric PK studies, optimal design-based approaches could be viewed as an ethical requirement to minimise the number of children and the number of samples required.^32^ One disadvantage is that optimal design relies heavily on the proposed model being useful for the target population. Since many PK studies are exploratory, it may not be possible to define an adequate model a priori. Therefore, study design should include techniques such as simulation re-estimation studies (to test whether a proposed design can recapture known parameters) and ensure samples covering the whole dosing interval are included at the population level, if not in every patient. Solely opportunistic sampling can give poor model parameter estimates, and when feasible obtaining samples at rational time points should be coordinated with routine blood tests.^34^ When samples cannot be optimally timed, the so-called ‘scavenged’ sampling methods may be adopted, provided the analytes being studied are suitable.^35^

### Recommendations for research in practice

We recommend incorporation of the steps summarised in table 3 when designing paediatric and neonatal PK studies to maximise study quality and the feasibility of developing new evidence-based dosing recommendations. Incomplete reporting of PK studies hinders the utility and comparability of study results, as highlighted by the 2015 consensus-based ClinPK statement.^36^ The ClinPK minimum reporting criteria will most likely benefit from adaptation to address the unique challenges of paediatric/neonatal PK study design, warranting future research.

**Table 3:** Steps to planning paediatric and neonatal pharmacokinetic studies

Protocol development	The study team receives input from suitably trained expert(s), including paediatric/neonatal clinical pharmacologists, analytical chemists and pharmacometricians (with expertise in popPK modelling and statistics).
Study design	The suitability and feasibility of optimal design is assessed. Consider the potential influence of formulation on PK studies designed to optimise dose.
Sample size	The sample size calculation is informed by ‘optimal design’. The target age range(s) of study participants and the number of participants in each age group are determined. In preterm neonatal PK studies, investigators define how many subgroups of prematurity are included (usually banded by gestational age at birth and postnatal age).
Sampling plans	The number of samples needed per participant and their timing (optimum timing vs opportunistic, or a combination) is justified. Acceptable method(s) of sampling is defined.
Patient involvement	Determine if there is a role for parent/child involvement in study design.
Analytical chemistry	Preferred matrix is confirmed (eg, blood vs serum or other biospecimen) and whether total or unbound concentrations will be measured. Stability of analytes in this matrix is verified (short-term and long-term, in relevant storage conditions).
Pharmacometrics	Input from popPK statisticians is used for design questions above, and to plan which type of PK modelling is used and how to plan covariate analyses.
Implementation (1)	Discuss with formulary committee where feasible to verify quantity and quality of new PK/PD data required to update paediatric or neonatal dosing recommendations.
Implementation (2)	For larger, multicentre/international studies, liaison with the regulators can also be helpful to explore, for off-patent drugs, how much (new) paediatric/neonatal PK data would be required to change the labelling.

PD, pharmacodynamic; PK, pharmacokinetic; popK, population PK.

### Changing clinical practice: implementing evidence-based formularies

**Figure 1:**
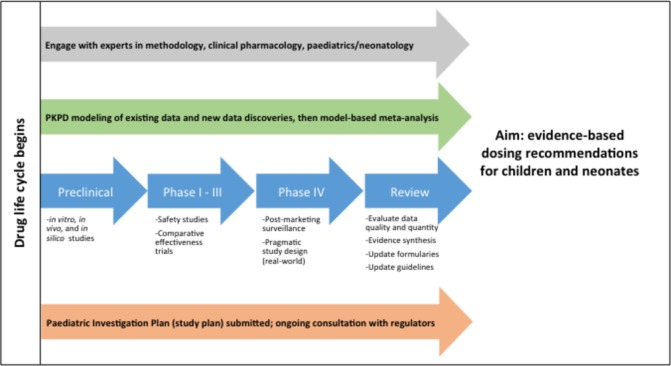
Paediatric product life-cycle from development to implementation. PD, pharmacodynamic; PK, pharmacokinetic.

Even when new PK studies are completed in children, these new data may be ignored by formulary committees and not used to inform future dosing recommendations. There are currently few transparent evidence-based paediatric and neonatal formularies, where the end users (ie, physicians or pharmacists) can easily access the evidence on which the dosing recommendations are based. The Dutch have reported their experience of developing and publishing an evidence-based formulary, with clear descriptions of the timelines and funding required.^37^ This example demonstrates the feasibility of such endeavours and paves the way for other national and international formularies to follow suit. It is our belief that incorporating the latest PK evidence into formularies should become routine to inform dosing at the bedside. Our vision for the life-cycle of paediatric therapeutics is summarised in figure 1.

### Current PK research needs

#### Funding and infrastructure for paediatric medicines research

While industry funds paediatric trials as requested by regulators, large public funders, including the NIH (National Institutes of Health), the EU FP7 programme and the Medical Research Council, have all recently funded paediatric PK research initiatives, as have charities such as the Paediatric European Network for the Treatment of AIDS and Infectious Diseases.^38^ While such influential bodies continue to prioritise paediatric medicines, these streams are invaluable to support investigator-led research. PK research into widely used, old, off-patent medicines is essential to facilitate modern dose optimisation strategies and often depends on clinical academics seeking public funding; these endeavours are supported by, for example, the FDA and the European Medicines Agency priority lists, which retain focus on improving the evidence base for paediatric medicines, despite changing political agendas.

Given the new requirements for PK data in all age groups for new drugs, industry-partnered approaches should strengthen paediatric PK research, through sharing knowledge, expertise and training opportunities. Funders should consider small pilot studies if required to design larger scale paediatric trials or where, for rare diseases when an adequately powered trial is unfeasible, research into using PK/PD surrogate endpoints as an alternative strategy is warranted. Separately, specific funding is needed for methodological aspects of PK/PD research including developmental pharmacology.

To date, trial conduct is often hampered by inadequate infrastructure to support study delivery. Physicians may be unconvinced of the need for paediatric PK studies, given perceived research burdens. Worldwide, most paediatric hospitals lack high-quality, Good Clinical Practice (GCP)-compliant infrastructure. Educating all paediatric healthcare professionals on the urgent need for such trials is of utmost importance.

For cost-effectiveness, specialist networks fostering and disseminating existing expertise are highly beneficial. International initiatives such as the Global Research in Paediatrics (http://www.grip-network.org)^39^ and the new International Neonatal Consortium (https://c-path.org/programs/inc/) are addressing the scarcity of expertise in paediatric clinical pharmacology. Sustainability will depend on adequate expertise and the training of future researchers. It is also important to recognise the major contributions of collaborative paediatric clinical trial networks, such as the National Institute for Health Research Clinical Research Network: Children, the Dutch Medicines for Children Research Network, Canada’s KidsCAN Trials and the Paediatric Trials Network.^40^ The recently launched EU-funded Horizon PEDCRIN project aims to establish a European Paediatric Clinical Trial Network, moving towards a continent-wide infrastructure. The continued funding of such dedicated networks will provide a solid infrastructure to underpin future activities.

#### Research agenda

An internationally embraced research agenda for paediatric PK is needed. All stakeholders including clinicians, researchers, policy makers, funders, patients and families should play a role in defining this. While no formal agenda exists, the current literature highlights knowledge gaps and areas requiring further development. The most urgent are the following:

- Increasing the number of paediatric studies collecting PK data.
- Evaluating the risk–benefit profile for conducting efficacy studies without prior PK data, including ethical considerations.
- Understanding renal function markers, and scaling renal function, identifying markers without the limitations of creatinine, for example, cystatin-C,^26^ and identifying new urinary biomarkers which, in addition to denoting drug-induced kidney injury, may provide useful markers of impaired renal drug clearance.
- Promoting standardised minimum data recommendations and data sharing between researchers and formulary committees to facilitate evidence-based dosing recommendations and timely updates across borders.
- Funding agencies should mandate a paediatric pharmacologist reviewer for all drug efficacy study protocols.

## Conclusions

Although undertaking high-quality paediatric PK studies is challenging, the tools and expertise needed are now available and affordable. Model-based approaches address many obstacles in PK study design and delivery. Thus, there is a unique opportunity to drive paediatric medicines research forward, providing adequate research infrastructure is developed and sustained. Political and practical support from regulators and physicians is paramount to the future success of these initiatives. As paediatric PK data continue to improve, we must create mechanisms for incorporating regular, rigorous reviews of the latest data into routine formulary updates, to ensure evidence-based dosing can be implemented to support rational paediatric pharmacotherapy. The importance of parallel paediatric PD research must not be forgotten, which we discuss in a related review.^41^ However, with the current energy and momentum in this field, it will be feasible to dramatically improve the evidence supporting optimal dosing of medicines for children in the near future, with great benefits for our patients.
